# Transcription start sites at the end of protein-coding genes

**DOI:** 10.1186/s40246-018-0146-6

**Published:** 2018-03-16

**Authors:** Ming-Yu Huang, Ji-Long Liu

**Affiliations:** College of Veterinary Medicine, South China Agricultural University, Guangzhou, China

**Keywords:** Transcriptional readthrough, Downstream of gene-containing transcripts (DoGs), TSS-seq

## Abstract

Previous studies demonstrated that massive induction of transcriptional readthrough generates downstream of gene-containing transcripts (DoGs) in cells under stress condition. Here, we analyzed TSS-seq (transcription start site sequencing) data from the DBTSS database. We investigated TSS tags at the end of gene for all pan-stress and untreated-cell DoGs, in comparison with expression-matched non-DoGs. We observed significantly more TSS tags at the end of pan-stress and untreated-cell DoG genes than non-DoG genes, even though their TSS tags in the promoter is the same. Importantly, the median value of TSS tags at gene end normalized to gene promoter is significantly higher than the median expression ratio of short DoG to host gene and of long DoG to host gene. Our results indicate that downstream overlapping long non-coding RNAs derived from the TSS at the gene end may be an important source of DoGs.

**Electronic supplementary material:**

The online version of this article (10.1186/s40246-018-0146-6) contains supplementary material, which is available to authorized users.

## Background

Vilborg et al. analyzed nuclear transcriptome changes in SK-N-BE(2)C human neuroblastoma cells [1] and NIH3T3 mouse fibroblast cells [2] under heat shock, osmotic stress, and oxidative stress by using RNA-seq. They observed massive induction of transcriptional readthrough, or downstream of gene-containing transcripts (DoGs), under all stress conditions. Being long (often > 45 kb) and diverse (> 2000 species), DoGs may contribute significantly to the transcriptome.

Previously, we have demonstrated that the progesterone receptor (PGR) gene processes a very long 3′-UTR of approximately 10 kb and this length can be further extended in the monkey endometrium from the view of sequencing data [3]. However, we have found that this extension is not due to a readthough, but an independent transcription start site (TSS) at the end of PGR, resulting a sense long non-coding RNA (lncRNA) overlapping with PGR 3′-UTR. Thus, we questioned whether these DoGs observed by Vilborg et al. [1, 2] are downstream overlapping lncRNAs instead of readthrough products from the promoter of protein-coding genes.

To answer this question, we performed a bioinformatic analysis of the public data. Our preliminary results challenge the readthough model proposed by Vilborg et al. [1, 2].

## Methods

The TSS-seq data performed on NIH3T3 cells were downloaded from the DataBase of Transcriptional Start Sites (DBTSS, https://dbtss.hgc.jp). The DNaseI data for NIH3T3 cells as well as Pol2, H3K4m1, and H3K4m3 for MEF (mouse embryo fibroblast) cells were derived from the ENCODE project (https://www.encodeproject.org). The UCSC Genome Browser (http://genome.ucsc.edu/) was used to display TSS-seq data and chromatin features for four representative DoGs: Hnrnpa2b1, Txn1, Hspa8, and Ifitm2. The genomic coordinates were based on mouse mm9 genome assembly.

In addition to the four representative DoGs, we extracted the genomic coordinates for all the DoGs described by Vilborg et al. [2]. The number of TSS tags at 1-kb region of a gene promoter and gene end were summarized according to TSS-seq data. Because DoGs and non-DoGs differ in size and gene expression levels, we constructed an equal size expression-matched subset for non-DoGs by randomly sampling using in-house PERL scripts. Difference between groups was tested by the nonparametric Mann-Whitney *U* test implemented in MATLAB (MathWorks, version 7.5).

**Table 1:** Statistical analysis of TSSs at gene end

Category	Type	Median (25th–75th quantiles)	*P* value
Promoter-1 k	Non-DoGs	204 (61–639)	
	Pan-stress DoGs	207 (47–782)	0.687
	Untreated DoGs	203 (78–718)	0.534
End-of-gene-1 k	Non-DoGs	15 (2–51)	
	Pan-stress DoGs	22 (5–75)	0.0000285*
	Untreated DoGs	23 (6–79)	0.00000276*
End-of-gene-1 kb/promoter–1 kb	Non-DoGs	0.0769 (0.0138–0.4071)	
	Pan-stress DoGs	0.1149 (0.0193–0.6251)	0.000289*
	Untreated DoGs	0.1279 (0.0191–0.7435)	0.0000193*

Expression-matched non-DoGs were randomly selected, and Mann-Whitney *U* test was performed. *P* values were calculated by comparing to non-DoGs

*P < 0.05

## Results and discussion

**Fig. 1:**
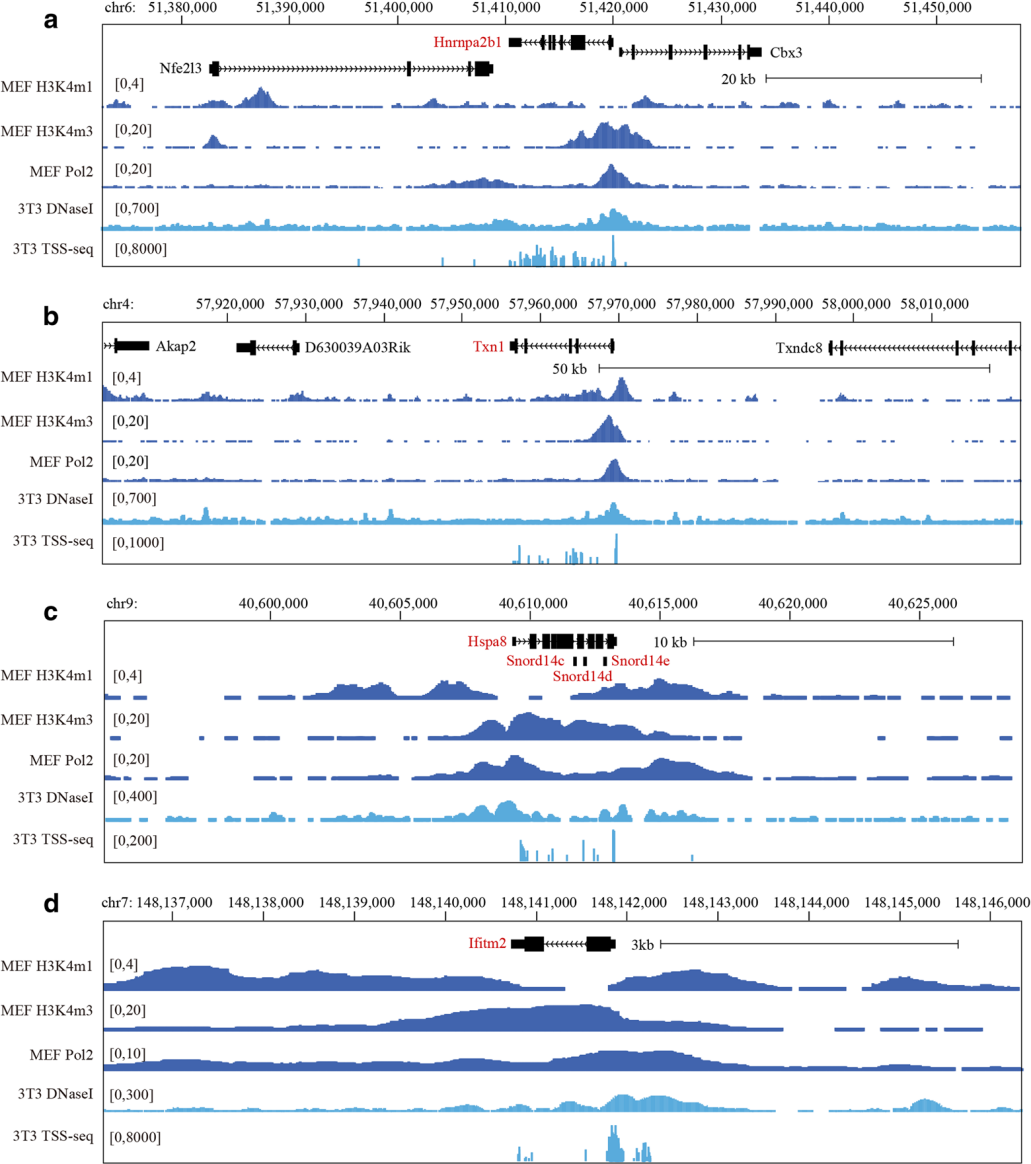
TSS-seq data and chromatin features for four representative DoGs. **a** Hnrnpa2b1. **b** Txn1. **c** Hspa8. **d** Ifitm2. Open chromatin in the genome is marked by Pol2 and DNaseI occupancy. H3K4me3 is a promoter marker and H3K4me1 is an enhancer marker

By combining oligo-capping with high throughput sequencing, the TSS-seq approach is able to collect genome-wide TSS information together with a quantitative analysis of the expression levels of transcripts [4]. We examined TSS-seq data performed on NIH3T3 cells from the DBTSS database [5]. For all four representative DoGs (Hnrnpa2b1, Txn1, Hspa8, and Ifitm2) [2], the number of TSS tags at the end of a gene is one order of magnitude lower than that at a promoter, except Hspa8 (Fig. 1). Hspa8 exhibits higher number of TSS tags at the gene end compared to the promoter, likely due to intronic snoRNAs. These TSSs may generate lncRNAs with an independent promoter at the gene end.

We next investigated TSS tags at the end of a gene for all pan-stress and untreated-cell DoGs, in comparison with expression-matched non-DoGs. We observed significantly more TSS tags at the end of pan-stress and untreated-cell DoG genes than those of non-DoG genes, even though their TSS tags in the promoter is the same. Furthermore, we normalized the number of TSS tags at the gene end to the number of TSS tags at the promoter of the same gene. Significance was also reached for the normalized data (Table 1 and Additional file 1: Figure S1).

Additionally, the median value of TSS tags at gene end normalized to gene promoter is 0.1088, slightly higher than the median expression ratio of short DoG to host gene (0.0146) and of long DoG to host gene (0.0067). These results indicate that TSSs at a gene end may be an important source of DoGs.

## Conclusion

Taken together, by analyzing TSS-seq data, we suggested that TSSs at the gene end may be an important major source of DoGs. Therefore, TSS-seq along with a large scale of Northern blot and tiling PCR experiments are required by Vilborg et al. [1, 2] to support their idea that most DoGs are continuous transcripts caused by a readthrough of protein-coding genes.

## Additional file

Additional file 1: **Figure S1.** Statistical analysis of TSSs at gene end (related to Table 1). (A) Number of TSS tags at 1-kb region of gene promoter and gene end, among pan-stress DoGs, untreated-cell DoGs, and non-DoGs. (B) Normalized number of TSS tags at gene end to the number of TSS tags at gene promoter, among pan-stress DoGs, untreated-cell DoGs, and non-DoGs. (TIFF 468 kb)

## Abbreviations

DoGs: Downstream of gene-containing transcripts
Hnrnpa2b1: Heterogeneous nuclear ribonucleoprotein a2/b1
Hspa8: Heat shock 70 kDa protein 8
Ifitm2: Interferon-induced transmembrane protein 1
lncRNA: Long non-coding RNA
PGR: Progesterone receptor
TSS: Transcription start site
Txn1: Thioredoxin 1

## References

[1] Vilborg A, Passarelli MC, Yario TA, Tycowski KT, Steitz JA (2015). Widespread inducible transcription downstream of human genes. Mol Cell.

[2] Vilborg A, Sabath N, Wiesel Y, Nathans J, Levy-Adam F, Yario TA, Steitz JA, Shalgi R. Comparative analysis reveals genomic features of stress-induced transcriptional readthrough. Proc Natl Acad Sci USA. 2017;114(40):E8362–E8371. 10.1073/pnas.1711120114 PMC5635911 28928151

[3] Liu JL, Liang XH, Su RW, Lei W, Jia B, Feng XH, Li ZX, Yang ZM (2012). Combined analysis of microRNome and 3′-UTRome reveals a species-specific regulation of progesterone receptor expression in the endometrium of rhesus monkey. J Biol Chem.

[4] Tsuchihara K, Suzuki Y, Wakaguri H, Irie T, Tanimoto K, Hashimoto S, Matsushima K, Mizushima-Sugano J, Yamashita R, Nakai K (2009). Massive transcriptional start site analysis of human genes in hypoxia cells. Nucleic Acids Res.

[5] Suzuki A, Wakaguri H, Yamashita R, Kawano S, Tsuchihara K, Sugano S, Suzuki Y, Nakai K (2015). DBTSS as an integrative platform for transcriptome, epigenome and genome sequence variation data. Nucleic Acids Res.

