# Supplementary figures and images for: Transcription start sites at the end of protein-coding genes
Source: Hum Genomics. 2018 Mar 16;12:15. doi: 10.1186/s40246-018-0146-6 (PMC5857071; doi:10.1186/s40246-018-0146-6)

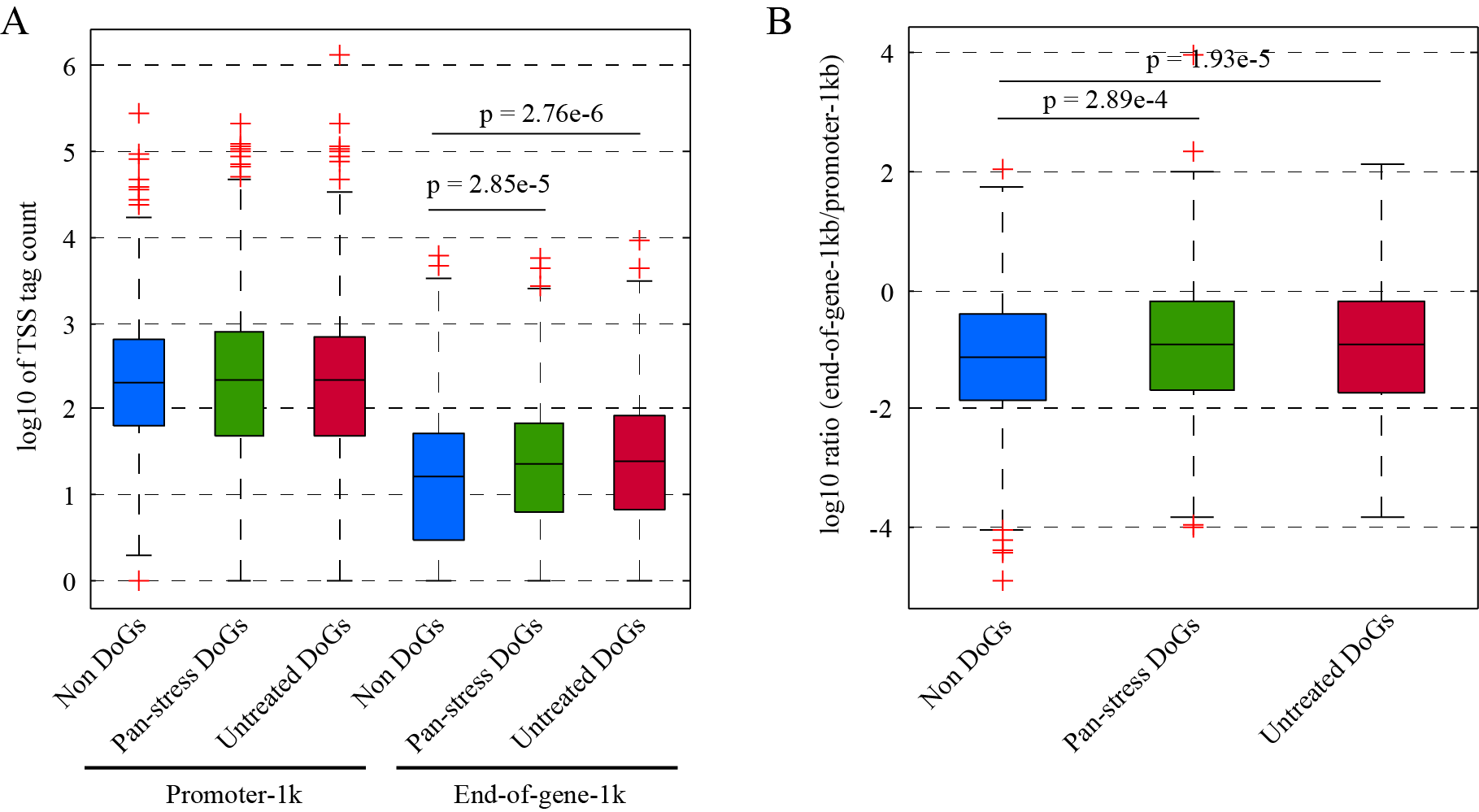

Supplement: Supplementary file 1 — Figure S1. Statistical analysis of TSSs at gene end (related to Table 1). (A) Number of TSS tags at 1-kb region of gene promoter and gene end, among pan-stress DoGs, untreated-cell DoGs, and non-DoGs. (B) Normalized number of TSS tags at gene end to the number of TSS tags at gene promoter, among pan-stress DoGs, untreated-cell DoGs, and non-DoGs. (TIFF 468 kb) [file 40246_2018_146_MOESM1_ESM.tif]
